# The JA–CsMYC2.1–CsNOMT–sakuranetin module contributes to differential anthracnose resistance in *Camellia sinensis*

**DOI:** 10.1093/hr/uhag022

**Published:** 2026-01-29

**Authors:** Xueying Zhang, Chaomin Chen, Linying Li, Yuqing He, Qinhua Lu, Da Li, Xuanyu He, Qingsheng Li, Gaojie Hong

**Affiliations:** State Key Laboratory for Quality and Safety of Agro-Products, Key Laboratory of Biotechnology in Plant Protection of MARA, Key Laboratory of Green Plant Protection of Zhejiang Province, Institute of Virology and Biotechnology, Zhejiang Academy of Agricultural Sciences, Hangzhou 310021, China; State Key Laboratory for Quality and Safety of Agro-Products, Key Laboratory of Biotechnology in Plant Protection of MARA, Key Laboratory of Green Plant Protection of Zhejiang Province, Institute of Virology and Biotechnology, Zhejiang Academy of Agricultural Sciences, Hangzhou 310021, China; State Key Laboratory for Quality and Safety of Agro-Products, Key Laboratory of Biotechnology in Plant Protection of MARA, Key Laboratory of Green Plant Protection of Zhejiang Province, Institute of Virology and Biotechnology, Zhejiang Academy of Agricultural Sciences, Hangzhou 310021, China; State Key Laboratory for Quality and Safety of Agro-Products, Key Laboratory of Biotechnology in Plant Protection of MARA, Key Laboratory of Green Plant Protection of Zhejiang Province, Institute of Virology and Biotechnology, Zhejiang Academy of Agricultural Sciences, Hangzhou 310021, China; Institute of Sericulture and Tea, Zhejiang Academy of Agricultural Sciences, Hangzhou 310021, China; Institute of Sericulture and Tea, Zhejiang Academy of Agricultural Sciences, Hangzhou 310021, China; State Key Laboratory for Quality and Safety of Agro-Products, Key Laboratory of Biotechnology in Plant Protection of MARA, Key Laboratory of Green Plant Protection of Zhejiang Province, Institute of Virology and Biotechnology, Zhejiang Academy of Agricultural Sciences, Hangzhou 310021, China; Institute of Sericulture and Tea, Zhejiang Academy of Agricultural Sciences, Hangzhou 310021, China; State Key Laboratory for Quality and Safety of Agro-Products, Key Laboratory of Biotechnology in Plant Protection of MARA, Key Laboratory of Green Plant Protection of Zhejiang Province, Institute of Virology and Biotechnology, Zhejiang Academy of Agricultural Sciences, Hangzhou 310021, China

## Abstract

Anthracnose, caused by *Colletotrichum* species, poses a significant threat to global tea (*Camellia sinensis*) production, yet its inducible resistance mechanisms remain largely uncharacterized. Through integrated transcriptomic and metabolomic analyses of the anthracnose-resistant cultivar ‘Zijuan’ and the susceptible cultivar ‘Longjing43’, we identified sakuranetin as a key phytoalexin in tea plants and elucidated a complete jasmonic acid (JA)-mediated defense pathway. Our functional characterization revealed that CsNOMT (Cha09g008790), a naringenin 7-O-methyltransferase, catalyzes sakuranetin biosynthesis with high substrate specificity. Following infection with *Colletotrichum camelliae*, sakuranetin accumulated exclusively in resistant cultivars, exhibiting superior antifungal activity compared to major tea catechins. Functional validation demonstrated that overexpression of CsNOMT enhanced both sakuranetin accumulation and disease resistance, while gene silencing compromised both traits. Mechanistically, we established that the JA-responsive transcription factor CsMYC2.1 directly activates *CsNOMT* transcription via G-box binding, establishing a novel JA–CsMYC2.1–CsNOMT–sakuranetin defense axis that distinguishes resistant from susceptible tea cultivars. This study represents the first comprehensive characterization of inducible phytoalexin-mediated immunity in tea, providing immediate applications for sustainable tea production. CsNOMT serves as a valuable functional marker for resistance breeding, while sakuranetin emerges as a promising natural biopesticide to reduce reliance on synthetic fungicides.

## Introduction

Tea (*Camellia sinensis*) is the second most consumed beverage globally and ranks among the most economically significant crops. However, its production is severely threatened by anthracnose, a disease caused by various species of *Colletotrichum camelliae* [1, 2]. These pathogens exhibit latent infection characteristics, overwintering as mycelium or spores in infected plant tissues, leading to yield losses of 30% to 50% due to wind or rain-dispersed spores [3–5]. The reliance on synthetic fungicides for disease management raises critical concerns regarding environmental contamination and chemical residues in tea products consumed as direct infusions [6–8]. This situation underscores the urgent need for sustainable alternatives by exploring and utilizing the plant's natural defense mechanisms.

Plants have evolved complex defense strategies, including the synthesis of phytoalexins, antimicrobial compounds produced in response to pathogen attacks [9–11]. Among these, sakuranetin (SAK) is a flavonoid-derived phytoalexin identified in rice. It accumulates in response to various stressors, including pathogen attacks, herbivore feeding, UV radiation, and hormonal signals, highlighting its significance in plant defense [12–15]. Recent studies demonstrate that SAK plays a critical role in plant immunity by exhibiting potent antifungal properties, which include inhibiting the intracellular phagocytosis of pathogens and suppressing endosymbiotic bacteria associated with insect pests [16, 17]. SAK is synthesized by the enzyme naringenin 7-O-methyltransferase (NOMT), which converts naringenin into SAK [14]. While both SAK and NOMT have been well characterized in rice for their roles in disease resistance, their functions in other plants, particularly tea, remain largely unexplored.

Jasmonic acid (JA) is a key signaling molecule in plant defense that activates the expression of numerous genes involved in phytoalexin biosynthesis and other defense responses [18–20]. Upon pathogen recognition, JA signaling cascades are initiated, leading to the activation of transcription factors that regulate the expression of critical biosynthetic genes [21, 22]. This regulatory cascade has been thoroughly studied in both model systems and various plant species, revealing that JA-responsive MYC2 transcription factors directly induce the expression of phytoalexin biosynthesis genes [19, 23, 24]. For instance, PpMYC2 strengthens fruit resistance by activating genes involved in lignin synthesis [25]. JA treatment or overexpression of FtCYP94C1, a gene homologous to AtMYC2 related pathways, enhances flavonoid accumulation and resistance, partially bypassing canonical JA signaling [26]. Despite the extensive characterization of JA-mediated defense responses across numerous plant species, the specific role of JA signaling in activating similar inducible defense mechanisms in perennial crops like tea, particularly the biosynthesis of phytoalexins in response to pathogens, remains poorly understood. While tea contains a diverse range of phytochemicals recognized for their roles in plant defense, much of the research has predominantly focused on constitutive defenses, such as catechins and caffeine [27–30]. Therefore, insights into JA-mediated inducible defenses are essential for developing effective resistance strategies and breeding cultivars that rely less on synthetic inputs, thereby enhancing the safety of tea products.

To address this knowledge gap and investigate the potential role of SAK in tea's defense against anthracnose, we performed integrated transcriptomic and metabolomic analyses comparing the anthracnose-resistant cultivar ‘Zijuan’ (ZJ) with the susceptible cultivar ‘Longjing43’ (LJ43). Our multi-omics approach revealed significant upregulation of genes associated with JA signaling and flavonoid biosynthesis pathways in ZJ, particularly several caffeic acid O-methyltransferase (COMT) genes. Further evolutionary analyses, combined with *in vitro* enzymatic assays and *in vivo* functional studies, demonstrated that a key tea COMT (Cha09g008790) functions analogously to OsNOMT in rice, catalyzing the synthesis of SAK. Therefore, we hypothesized that the differential activation of the JA signaling pathway and subsequent regulation of *NOMT* expression leads to contrasting levels of SAK accumulation, thereby conferring differing levels of resistance to anthracnose. By unraveling the molecular mechanisms underlying SAK's role in enhancing tea plant resistance to anthracnose, this research not only contributes to the understanding of plant immunity but also supports the development of sustainable agricultural practices and the improvement of tea quality.

## Results

### Global metabolic profiling between LJ43 and ZJ

**Figure 1:**
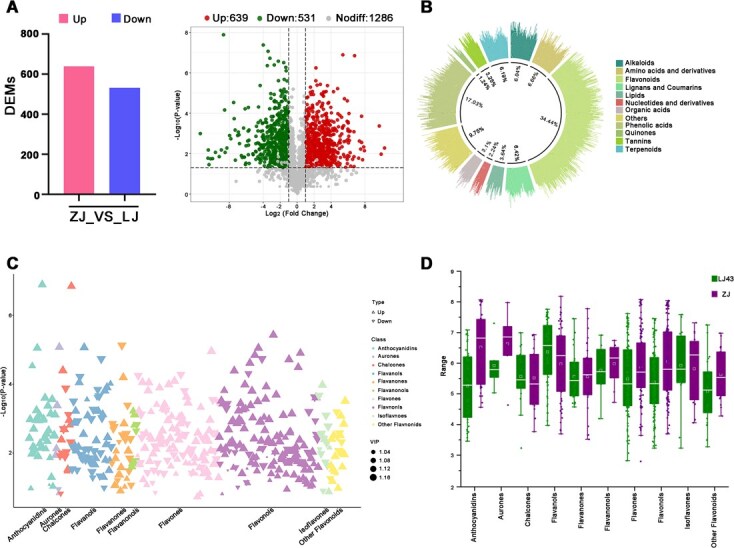
Metabolic profiling of tea cultivars LJ43 and ZJ. (A) Counts of different metabolites identified in ZJ compared with LJ43. Volcano plots representing metabolic changes in ZJ compared with LJ43. (B) Circle chart showing the proportion of primary metabolite classifications, with color-coding distinguishes metabolites categories. (C) Scatter plot depicting the classification of differentially accumulated flavonoid metabolites between ZJ and LJ43, with colors coding highlighting different subclasses of flavonoids. (D) Boxplot of flavonoid differences between LJ43 and ZJ tea cultivars, with the central line indicating the median.

We conducted targeted metabolic profiling on the leaves of genotypes LJ43 and ZJ, identifying a total 1956 metabolites. Principal component analysis (PCA) and correlation analysis revealed that biological replicates were more closely related within each genotype than between genotypes detected total of 1170 differential metabolites (DMs) were detected between the two genotypes, with 639 upregulated and 531 downregulated in ZJ compared to LJ43 (Fig. 1A, Table S1). Among these DMs, flavonoids constituted the largest group, accounting for 34.4% (Fig. 1B). Further classification of these flavonoid metabolites, as illustrated by volcano plots, divided them into 10 categories: anthocyanidins, aurones, chalcones, flavanols, flavanones, flavanonols, flavones, flavonols, isoflavones, and other flavonoids. In comparison to LJ43, most metabolites levels were elevated in ZJ, except for chalcones and flavanols (Fig. 1C and D). The purple foliage cultivar ‘ZJ’ is a somatic mutant derived from the Yunnan Daye cultivar (*Camellia sinensis* var. assamica (Mast.) Kitamura), noted for its higher anthocyanin content relative to other Chinese purple tea cultivars. The enhanced flavonoid accumulation observed in ZJ, especially the robust presence of anthocyanins, suggests a potential role in disease resistance mechanisms, particularly against anthracnose pathogens.

### Comparative transcriptome and metabolome analysis reveals JA signaling and flavonoid pathway activation in resistant cultivars

**Figure 2:**
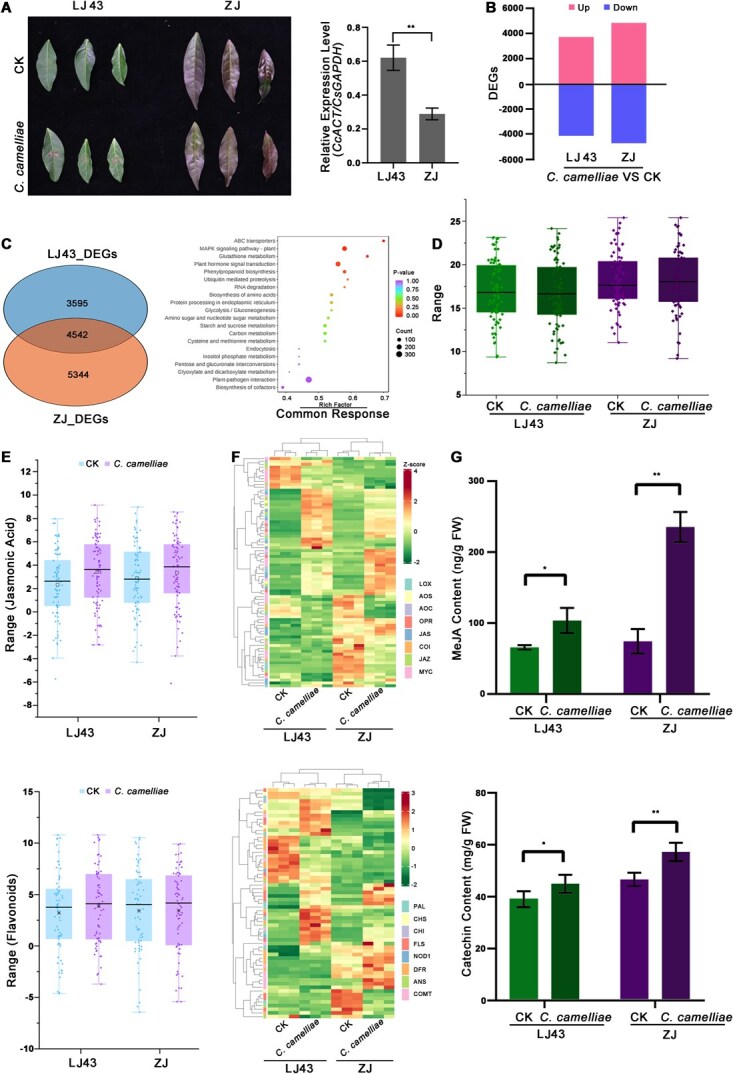
Transcriptional and metabolic responses of tea cultivars to *C. camelliae* infection. (A) Phenotypic differences in LJ43 and ZJ pre- and post-inoculation. Fungal content in infected leave of LJ43 and ZJ cultivars (*n* = 6 biological replicates). (B) Differentially expressed genes (DEGs) identified in LJ43 and ZJ cultivars before and after infection (|log2FC| > 1, FDR < 0.05). (C) Proportion of DEGs in LJ43 and ZJ cultivars. KEGG pathways commonly enriched among DEGs in both LJ43 and ZJ cultivars after *C. camelliae* infection. (D) Boxplot showing changes in flavonoid metabolite content in LJ43 and ZJ following *C. camelliae* infection. (E) Boxplot of JA and flavonoid pathway gene expression. (F) Heatmap of differential gene expression in JA and flavonoid pathways. (G) JA and catechin content in LJ43 and ZJ tea cultivars before and after anthracnose fungus treatment.

Disease assessment confirmed distinct resistance phenotypes, with ZJ showing restricted lesion development while LJ43 exhibited extensive pathogen spread, and quantitative analysis revealed significantly lower fungal content in infected ZJ leaves compared to LJ43 (Fig. 2A). Transcriptional profiling revealed that LJ43 showed 8137 DEGs following infection while ZJ exhibited 9886 DEGs, indicating more extensive transcriptional reprogramming in the resistant cultivar (Fig. 2B). Kyoto Encyclopedia of Genes and Genomes (KEGG) pathway enrichment analysis revealed significant associations with phenylpropanoid, flavonoid, MAPK signaling, and plant hormone signal transduction pathways (Fig. 2C and D). To further assess the expression of genes related to involved JA and flavonoids biosynthesis, boxplots were generated showing indicate the JA and flavonoid pathways were upregulated in response to anthracnose infection in both ZJ and LJ43. Targeted metabolic profiling found that following inoculation with the anthracnose fungus, the flavonoid content in the resistant variety ZJ was significantly upregulated compared to the susceptible variety LJ43, with higher fold changes in ZJ than in LJ43 (Fig. 2E and F). Subsequent detection of JA and catechin levels significant increases in tea leaves post-inoculation with anthracnose fungus. Notably, ZJ exhibiting more pronounced upregulation compared to LJ43, establishing enhanced JA-flavonoid signaling as a primary resistance mechanism (Fig. 2G).

### CsNOMT is preferentially induced in resistant cultivars and catalyzes SAK biosynthesis

**Figure 3:**
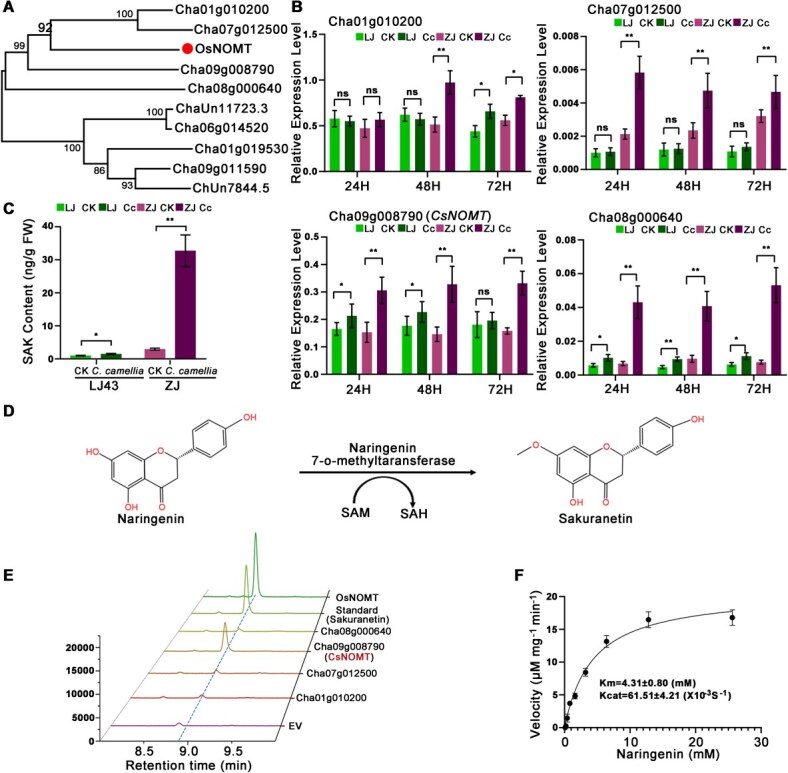
Characterization of COMT. (A) Phylogenetic tree of tea COMT and rice NOMT proteins. (B) Relative expression of four COMT genes in ‘LJ43’ and ‘ZJ’ after *C. camelliae* inoculation at 24, 48, and 72 hours. RT-qPCR data were introduced as mean values ± standard deviation (^**^P < 0.01; *n* = 3). (C) SAK levels in ‘LJ43’ and ‘ZJ’ after *C. camelliae* inoculation. (D) The final step in the biosynthetic pathway for SAK catalyzed by naringenin 7-O-methyltransferase (NOMT). SAM, S-adenosyl-l-methionine; SAH, S-Adenosyl-l-homocysteine. (E) LC–MS analysis of SAK produced by recombinant CsNOMT enzyme using naringenin as substrates. (F) Kinetic parameters for recombinant CsNOMT enzyme.

Given the prominent activation of flavonoid biosynthesis pathways, we focused on COMT genes showing significantly higher expression in ZJ than LJ43 (Fig. 2F). Evolutionary analysis revealed that COMT genes from tea plants share close evolutionary relationships with rice naringenin O-methyltransferase (NOMT) (Fig. 3A). Among four candidate CsCOMT genes, three showed significant upregulation in response to pathogen infection in both cultivars, with induction significantly stronger in ZJ than in LJ43 (Fig. 3B). LC–MS analysis revealed significant increases in SAK levels in ZJ tea leaves after anthracnose inoculation, whereas SAK content in LJ43 leaves remained stable (Fig. 3C). To assess enzymatic activity, we expressed recombinant proteins in *E. coli* and evaluated their ability to convert naringenin to SAK. Only the protein encoded by Cha09g008790 (designated CsNOMT) exhibited the expected catalytic function as confirmed by LC–MS analysis (Fig. 3D, E and Fig. S1). Kinetic characterization revealed a Km of 4.31 mM for naringenin and a Kcat of 61.51 × 10^−3^ s^−1^, indicating strong substrate affinity and high catalytic efficiency (Fig. 3F).

### SAK exhibits superior antifungal activity against *C. camelliae*

**Figure 4:**
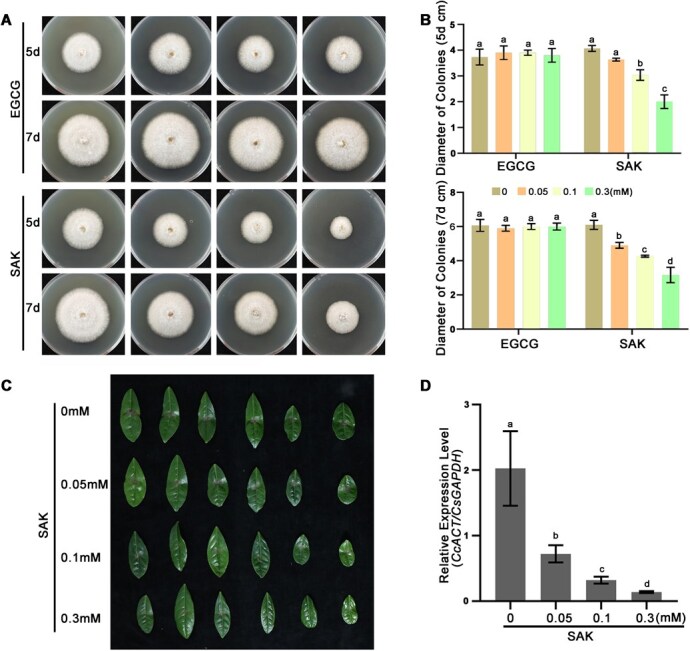
Antifungal activity of SAK against *C. camelliae*. (A, B) Inhibitory effects of SAK and epigallocatechin gallate (EGCG) on *C. camelliae* bacteria size. (C) Phenotypic changes in tea leaves of LJ43 and ZJ cultivars following SAK treatment and *C. camelliae* inoculation. (D) Relative expression levels of *C. camelliae* mRNA in tea leaves of LJ43 and ZJ cultivars following SAK treatment.

Having identified SAK as the enzymatic product of CsNOMT and a pathogen-inducible metabolite, we next investigated its direct antifungal properties. We compared SAK's efficacy against EGCG, which accounts for 70% to 80% of total tea polyphenol content. Low concentrations of EGCG (0.05 to 0.3 mM/mL) did not significantly affect *C. camelliae* mycelial growth, while SAK at just 0.05 mM/ml significantly inhibited mycelial growth (Fig. 4A and B). Exogenous application of various SAK concentrations (0 to 0.3 mM/mL) on tea plant leaves showed that higher concentrations significantly reduced necrotic lesions caused by fungal infection, with quantification of fungal biomass confirming marked decreases in anthracnose proliferation with increasing SAK concentration (Fig. 4C and D). These results demonstrate SAK's superior antifungal activity compared to major tea catechins.

### Functional validation confirms CsNOMT's role in disease resistance

**Figure 5:**
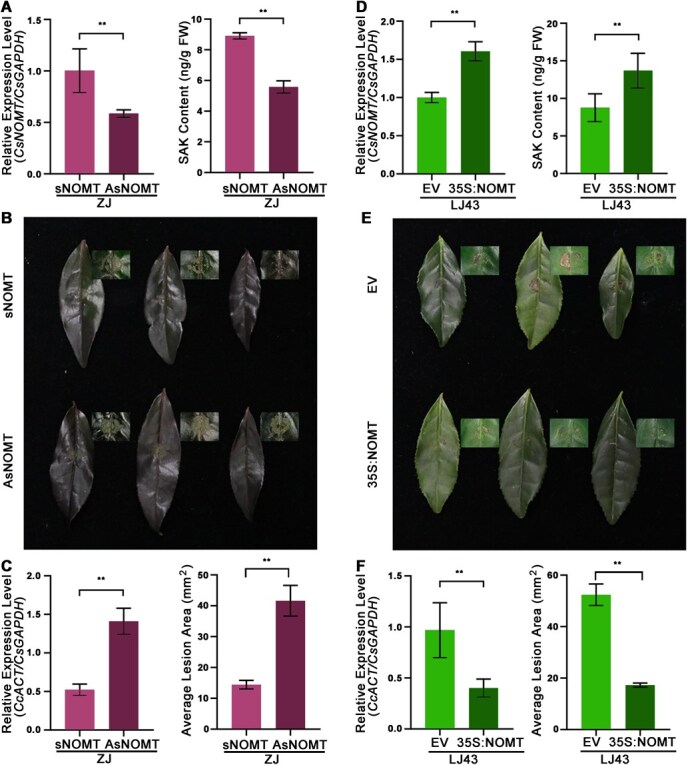
Functional analysis of *CsNOMT* in anthracnose resistance. (A) *CsNOMT* gene expression and SAK content in ZJ tea cultivars after sense *CsNOMT* (*sNOMT*) oligonucleotide and antisense *CsNOMT* (*AsNOMT*) oligonucleotide treatment. (B) Phenotypic changes in tea leaves after *CsNOMT* silencing and *C. camelliae* inoculation. (C) Relative expression levels of *C. camelliae* mRNA and average lesion area was calculated in tea leaves after *CsNOMT* silencing. (D) *CsNOMT* gene expression and SAK content after transient overexpression in LJ43 cultivars. (E) Phenotypic changes in tea leaves after *CsNOMT* overexpression and *C. camelliae* inoculation. (F) Relative expression levels of *C. camelliae* mRNA and average lesion area was calculated in tea leaves of LJ43 cultivars after transient overexpression of the *CsNOMT* gene and inoculation with *C. camelliae*.

To establish the causal relationship between *CsNOMT* expression, SAK accumulation, and disease resistance, we performed complementary gain- and loss-of-function analyses. Gene silencing experiments using antisense oligodeoxynucleotide (AsODN) technology in ZJ showed significantly reduced *CsNOMT* transcript levels after 24 hours compared to scrambled controls (Fig. 5A). This transcriptional suppression was associated with a corresponding 30% to 40% decrease in SAK accumulation and significantly larger lesion areas, indicating enhanced susceptibility (Fig. 5B and C). Conversely, transient transformation to overexpress *CsNOMT* in LJ43 leaves resulted in significant increases in *CsNOMT* expression levels, with SAK content elevated by approximately 30% compared to empty vector controls (Fig. 5D). After *C. camelliae* infection, leaves overexpressing *CsNOMT* exhibited smaller lesion areas than control groups (Fig. 5E and F). These complementary results establish CsNOMT-mediated SAK production as a key determinant of anthracnose resistance in tea.

### JA-responsive CsMYC2.1 directly regulates *CsNOMT* expression

**Figure 6:**
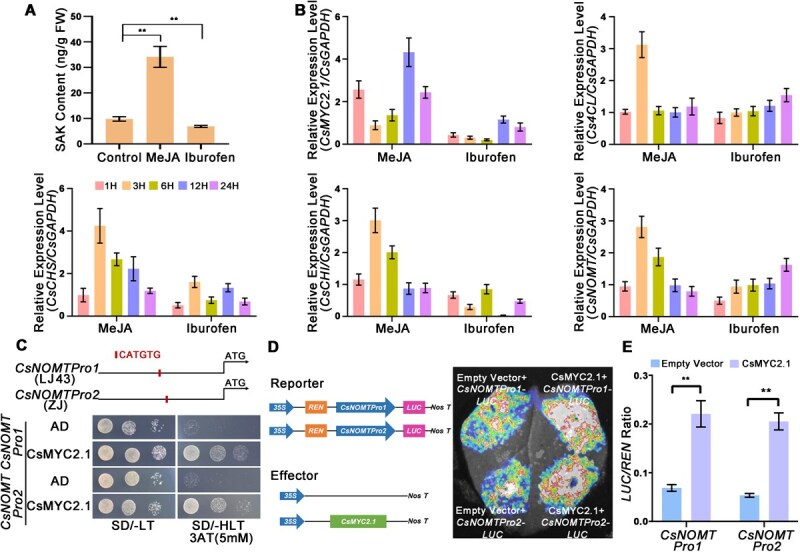
JA-responsive CsMYC2.1 directly regulates *CsNOMT*-mediated SAK biosynthesis. (A) SAK accumulation in tea leaves after exogenous MeJA treatment and JA biosynthesis inhibitor treatment. (B) Relative expression of *CsMYC2.1* and SAK biosynthetic genes (*Cs4CL*, *CsCHS*, *CsCHI*, and *CsNOMT*) under MeJA and JA biosynthesis inhibitor treatment. (C) Yeast One-Hybrid assay confirm binding of CsMYC2.1 to the *CsNOMTPro1* derived from either the susceptible (LJ43) or *CsNOMTPro2* from resistant (ZJ) tea cultivars. (D, E) Dual-LUC reporter assay demonstrated the ability of CsMYC2.1 transcriptional activation of the *CsNOMTPro1/2*. Relative firefly LUC to REN ratios in tobacco leaves, error bars indicate the SD of three biological replicates.

With the functional importance of CsNOMT established, we investigated the upstream regulatory mechanisms controlling its differential expression. Among four CsMYC2 transcription factors identified in the JA signaling pathway, only *CsMYC2.1* in ZJ was significantly induced upon *Colletotrichum* inoculation compared to LJ43 (Fig. S2) [23]. Exogenous JA treatment significantly induced SAK accumulation in tea leaves, with concentrations increasing by 2.4-fold, while JA biosynthesis inhibitor ibuprofen (Ibu) treatment reduced SAK levels by 31% (Fig. 6A). RT-qPCR analysis revealed that key SAK biosynthesis genes, including *Cs4CL*, *CsCHS*, *CsCHI*, and *CsNOMT*, were upregulated in methyl jasmonate (MeJA)-treated leaves, while inhibitor treatment suppressed their expression by 30% to 40% (Fig. 6B). MeJA treatment can significantly induce the synthesis of SAK in the leaves of LJ43 tea plants, thereby enhancing their resistance to *C. camelliae*. In contrast, treatment with the Ibu inhibits the synthesis of SAK in the leaves of Zijuan (ZJ) tea plants, resulting in decreased resistance (Fig. S3). Promoter analysis revealed that *CsNOMT* promoters from both cultivars contain conserved G-box elements (5′-CATGTG-3′). Yeast One-Hybrid and Dual-Luciferase assays confirmed that CsMYC2.1 directly binds to *CsNOMT* promoters and activates their expression (Fig. 6C).

**Figure 7:**
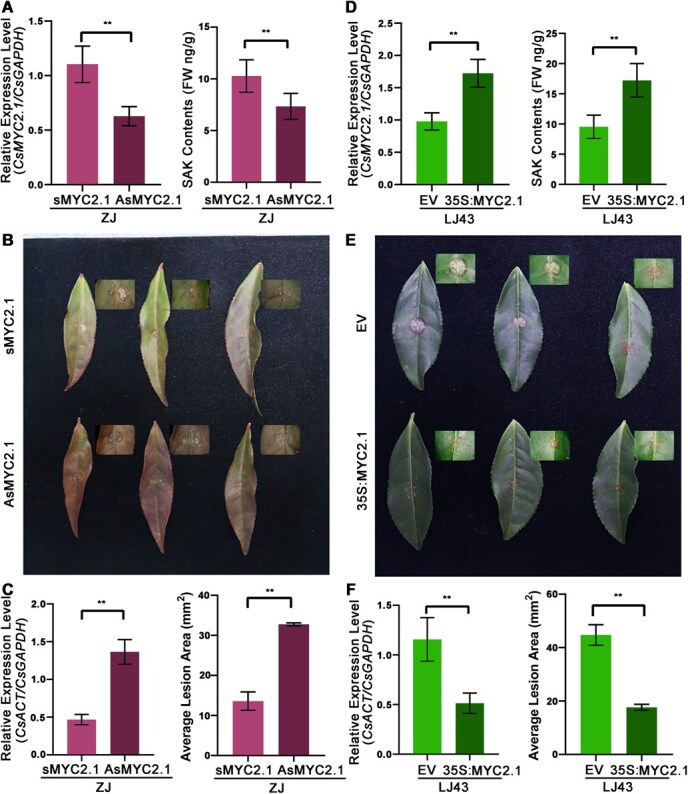
Functional characterization of *CsMYC2.1* in anthracnose resistance. (A) *CsMYC2.1* gene expression and SAK accumulation in ‘ZJ’ cultivars following sense *CsMYC2.1* (*sMYC2.1*) oligonucleotide and antisense *CsMYC2.1* (*AsMYC2.1*) oligonucleotide treatment. (B) Leaf phenotypes post *CsMYC2.1* silencing and *C. camelliae* infection. (C) Relative expression levels (*C. camelliae*) and average lesion area was calculated in tea leaves after *CsMYC2.1* silencing. (D) *CsMYC2.1* gene expression and SAK levels in ‘LJ43’ cultivars after transient overexpression. (E) Leaf phenotypes in *CsMYC2.1* overexpression and *C. camelliae* inoculation. (F) Relative expression levels (*C. camelliae*) and average lesion area was calculated in tea leaves of LJ43 cultivars after transient overexpression of the *CsMYC2.1* gene and inoculation with *C. camelliae*.

Importantly, the transcriptional activation capacity of CsMYC2.1 showed no significant difference between LJ43 and ZJ promoters, indicating that differential *CsNOMT* activation results from upstream JA signaling differences rather than promoter polymorphisms (Fig. 6D and E). This finding suggests that enhancing JA perception or signaling components could improve resistance in susceptible cultivars. Complementary functional analyses revealed that *CsMYC2.1* silencing reduced its expression by 43% and decreased SAK accumulation by 28.6%, while overexpression elevated expression by 76% and increased SAK by 80% (Fig. 7A and D). Silenced plants displayed larger lesion areas indicating enhanced susceptibility (Fig. 7B and C), whereas overexpression conferred resistance through reduced lesion development compared to controls (Fig. 7E and F). Collectively, our findings establish a complete JA–CsMYC2.1–CsNOMT–SAK defense axis that contributes significantly to anthracnose resistance in tea plants. This pathway represents a previously uncharacterized defense mechanism in tea and provides valuable targets for breeding and improving disease resistance in economically important tea cultivars.

## Discussion

Inducible phytoalexin-mediated resistance is a cornerstone of plant immunity, extensively characterized in model organisms like *Arabidopsis* (camalexin) and soybean (glyceollins, soyasaponins) [31–33]. However, the mechanisms underlying this crucial defense strategy remain poorly understood in economically vital perennial crops. Here we provide the first comprehensive characterization of SAK's defensive role in tea (*Camellia sinensis*), significantly advancing our understanding of perennial crop immunity. Although SAK's function is well documented in monocots like rice, our findings represent a significant breakthrough by demonstrating that this sophisticated defense paradigm is conserved and critically important in long-lived woody perennials [12, 14].

Our research establishes SAK as a potent, inducible antimicrobial agent that rapidly accumulates upon anthracnose infection. This dynamic induction profile fundamentally distinguishes it from tea's well-characterized constitutive defenses, such as catechins and caffeine [27–29]. Critically, our metabolomics analysis confirm caffeine is not pathogen induced and thus not a phytoalexin, despite its reported antibacterial properties [34]. Similarly, cyaniding-3-O-galactoside, although elevated in resistant cultivar ZJ, lacks typical inducible characteristics, and although JA signaling activation occurs during anthracnose infection in tea plants during anthracnose infection, intriguingly, exogenous JA application intriguingly fails to induce the anthocyanin-associated pink ring symptom [6]. Contrasting with tea's well-known constitutive defenses, SAK synthesis is triggered specifically by pathogen attack, positioning it as a frontline, inducible defense compound. Research has found that pathogen infection, brown plant hopper feeding, UV irradiation CuCl2, Plant hormones jasmonic acid and brassinolide can induce sakuranin accumulation in rice leaves [12, 14–16]. To address this metabolite consistently elevated across diverse resistant germplasms, we quantified SAK in three resistant (‘Zhongcha 108’, ‘Fuyun 6’, ‘Yingshuang’) and three susceptible (‘Huangguanyin’, ‘Zhenong 117’, ‘Wuniuzao’) cultivars post-*Colletotrichum inoculation*. Strikingly, no significant SAK variation was observed (*P* <0 .01), suggesting its limited universality as a defense marker (Fig. S4). *In vitro* assays have demonstrated that SAK exhibits superior antifungal potency (IC_50_ = 28.63 μg/mL vs. 8000 μg/mL for EGCG), which underscores its critical role in tea's immune arsenal and validates its potential as a natural antimicrobial agent [34].

Our work expands the functional understanding of tea's COMT gene family beyond traditional roles. While previous studies focused on their roles in secondary metabolism like catechin methylation [35, 36], we unveil a previously overlooked function in inducible immunity. Through phylogenetic analysis, we identified CsNOMT as the key biosynthetic gene, sharing evolutionary conservation with rice OsNOMT yet exhibiting tea-specific adaptations. The enzyme’s efficient kinetics (Km = 4.31 mM, kcat = 61.51 × 10^−3^ s^−1^) are optimally suited for rapid synthesis during pathogen attack. Crucially, our genetic evidence—from overexpression enhancing resistance to gene silencing compromising it establishes a direct causal relationship: CsNOMT expression directly dictates SAK levels, which in turn determines resistance outcomes.

Jasmonate (JA) signaling plays a pivotal role in activating defense responses, including the reprogramming of defense metabolism [22]. This pathway promotes the biosynthesis of diverse compounds, such as flavonoids and hydroxycinnamic acid derivatives, enhancing plant resistance against pathogens and herbivores [16, 37, 38]. Key molecular mechanisms linking broad defense signaling to specific phytoalexin production are evident. For instance, the ET/JA and MPK3 pathways converge on the transcription factor ERF1 to positively regulate camalexin biosynthesis in response to *B. cinerea* [33]. Similarly, JA treatment or *FtCYP94C1* overexpression (an *AtMYC2*-associated gene) enhances flavonoid accumulation and resistance in buckwheat [26]. Rice research has firmly established OsNOMT as the enzyme responsible for SAK biosynthesis, with evidence strongly supporting its regulation via the JA–MYC2 pathway [17, 19]. This mechanistic framework strongly parallels our findings on *Camellia sinensis* anthracnose resistance. We specifically observed significant upregulation of JA signaling in the resistant cultivar ‘ZJ’ following *Colletotrichum* infection. This activation induces the expression of *CsMYC2.1*, the core downstream transcriptional regulator of JA signaling. Crucially, CsMYC2.1 directly drives the expression of *CsNOMT*, leading to SAK accumulation and conferring the superior anthracnose resistance phenotype observed in ‘ZJ’. Regarding the important roles of salicylic acid (SA) in plant resistance to pathogens, we have also focused on the SA content and the expression levels of key genes involved in SA metabolism (such as *CsICS*) in ZJ and LJ43 [31, 38]. The results shown no significant differences in SA content and the expression of *CsICS1* before and after inoculation in the two varieties (Fig. S5). Our study demonstrates that tea resistance to anthracnose is primarily mediated by quantitative variation in JA–SAK signaling rather than SA pathway dominance or CsNOMT/CsMYC2.1 genetic polymorphisms, revealing a novel perennial-specific defense regulation mechanism.

**Figure 8:**
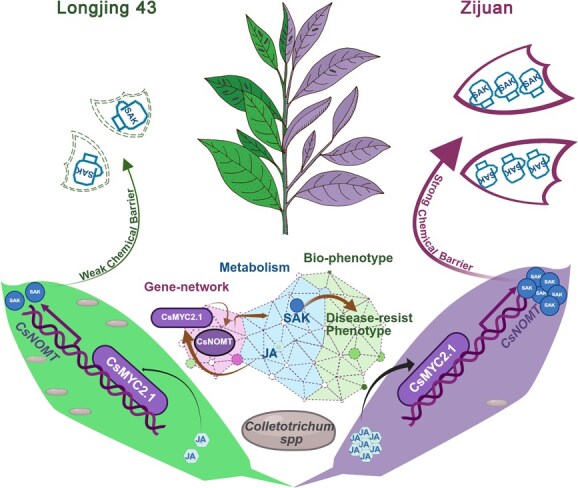
Proposed model for JA-mediated SAK-dependent anthracnose resistance. Upon *C. camelliae* infection, pathogen attack triggers differential JA signaling responses between tea cultivars. In the resistant cultivar Zijuan (ZJ), enhanced JA accumulation leads to stronger activation of the transcription factor CsMYC2.1, which directly binds to the *CsNOMT* promoter and activates its expression. Elevated *CsNOMT* expression drives increased SAK biosynthesis through the methylation of naringenin. The accumulated SAK acts as a potent phytoalexin, providing enhanced antifungal activity against anthracnose pathogens and contributing to the disease-resistant phenotype. In contrast, the susceptible cultivar Longjing 43 (LJ43) exhibits weaker JA signaling, resulting in lower CsMYC2.1 activation, reduced *CsNOMT* expression, minimal SAK accumulation, and consequently increased susceptibility to anthracnose infection. This model illustrates how cultivar-specific differences in JA signaling efficiency determine the strength of the CsMYC2.1–CsNOMT–SAK defense axis, ultimately controlling disease resistance outcomes in tea plants.

Through comprehensive functional analysis, we developed a unified model explaining cultivar-specific resistance patterns (Fig. 8). In this model, we dissected the complete regulatory architecture governing this defense, revealing the linear JA–CsMYC2.1–CsNOMT signaling cascade. This discovery demonstrates that resistance is not determined by structural polymorphisms in CsNOMT but rather by the plasticity and strength of upstream JA signaling. In resistant cultivars ZJ, robust JA responses trigger strong CsMYC2.1 activation, which then drives high-level CsNOMT transcription and massive SAK accumulation. The quantitative correlation between CsMYC2.1/CsNOMT expression levels and resistance phenotypes suggests these genes could serve as molecular markers for marker-assisted selection, potentially enhancing breeding efficiency through preliminary screening of germplasm collections. This finding shifts the paradigm for resistance breeding from gene structure to regulatory efficiency.

These findings have direct applications for sustainable tea production. The identification of SAK as an antimicrobial compound, combined with our understanding of the underlying genetic mechanisms, opens new avenues for crop improvement. Given the rising concerns surrounding the efficacy of synthetic fungicides and the emergence of resistant pathogens [39, 40], tea employs multifaceted strategies to balance defense and growth [41, 42]. Our findings advocate for innovative approaches to disease management. The potential development of biopesticides derived from SAK, alongside advancements in engineered Saccharomyces cerevisiae [43], offers valuable natural alternatives to chemical fungicides.

In conclusion, our findings illustrate that the principles of inducible phytoalexin-mediated immunity apply to woody perennials like tea, highlighting the role of JA signaling in resistance mechanisms. We demonstrate for the first time that SAK functions as a critical defensive compound in tea, and elucidate the complete JA–CsMYC2.1–CsNOMT–SAK pathway underlying anthracnose resistance. Beyond advancing our mechanistic understanding of plant immunity, this work provides practical applications for tea improvement, including potential molecular markers for breeding and SAK as a natural antimicrobial agent. Together, these findings establish a foundation for developing more resilient and sustainable tea cultivars through both genetic and chemical approaches.

## Materials and methods

### Plant materials and growth conditions

Two 2-year-old tea (*Camellia sinensis*) cultivars were chosen from the Zhejiang Academy of Agricultural Sciences: the anthracnose-resistant ‘Zijuan’ (ZJ) and the anthracnose-susceptible ‘Longjing 43’ (LJ43). Healthy plants with uniform growth were cultivated under regulated conditions, with a constant temperature of 25°C and a photoperiod of 14-hour light/10-hour dark.

### Pathogen inoculation and disease assessment

*Colletotrichum camelliae* inoculation was carried out according to a previously reported protocol [28]. Fungal spores obtained after incubation were resuspended in sterile distilled water, with the final concentration of the spore suspension calibrated to 1 × 10^6^ spores/ml using a hemocytometric counting. Tea leaves were fully expanded, surface-sterilized with 75% ethanol, rinsed with sterile water, and injected with 50 μl of spore suspension on the sterile water. An equivalent volumes of sterile water was applied to control leaves. Inoculated branches were placed in sterile water-filled containers, covered with humidified plastic bags, and incubated at 25°C. Photographs of the disease resistance phenotype of infected leaves were taken and assessed 5 days post-inoculation (dpi) (≥20 leaves per genotype). Fungal biomass was quantified by DNA-based quantitative PCR (qPCR), with tea GAPDH serving as the reference gene.

### RNA extraction and gene expression analysis

Total RNA isolation from leaf tissues employed RNAiso Plus reagent (Takara, Japan). The PrimeScript RT Kit (Takara) was then used to synthesize first-stand cDNA. SYBR Premix ExTaq II (Takara) on an ABI 7900HT platform (Applied Biosystems) to perform reverse transcription quantitative PCR analysis. Target gene expression levels, normalized against the endogenous control GAPDH and assessed using the 2^−ΔΔCt^ technique using primers specified in Table S2. Triplicate reactions were carried out.

### Metabolite extraction and LC–MS/MS analysis

Fresh samples (0.1 g) were crushed in liquid nitrogen and extracted overnight at 4°C in methanol after sonication (100 Hz/30 min). Widely targeted metabolite profiling and analysis were conducted by Metware (Wuhan, China). The supernatant (700 μl) from dual centrifugation (13 000 rpm/20 min) and 0.22 μm filtration was tested for catechin, MeJA, and SAK using LC–MS/MS (SCIEX Triple Quad 5500+). Method based on Zhao *et al.* [44].

### Sequence analysis and heterologous protein expression and purification

Full-length CsNOMT amplified from cDNA and cloned into pMD19-T. ClustalW and MEGA-X were used to align the sequences and determine the phylogeny, respectively.

His-tagged CsNOMT was expressed in *E. coli* BL21 using the pCold vector. Induced by 1 mM IPTG (OD_600_ = 0.6–0.8), purified with Ni-NTA beads (Qiagen, Germany). Enzymatic reactions: 50 μl of crude enzyme, 10 μl of 20 mM naringenin, 50 μl of 4 mM SAM, 50 μl of glycine–NaOH buffer (pH 9.5, 4 mM EDTA, 200 mM dithiothreitol) at 30°C for 2 hours. SAK detected by LC–MS/MS. Kinetic constants derived from naringenin gradients (0.1–20 mM). Non-linear regression analysis was used to determine the catalytic constants (Kcat) and Michaelis–Menten constants (Km).

### Enzymatic activity assays

Enzyme activity assays were performed in a reaction mixture containing 50 μl crude enzyme, 10 μl 20 mM naringenin in DMSO, 50 μl 4 mM S-adenosylmethionine (SAM), and 50 μl 400 mM glycine–NaOH buffer (pH 9.5, 4 mM EDTA, 200 mM dithiothreitol). Reactions were conducted at 30°C for 2 hours and terminated by adding 30 μl HCl (1 M). Methanol-diluted supernatants were analyzed by LC–MS/MS to determine SAK content. Kinetic parameters were determined using varying concentrations of naringenin (0.1–20 mM) with a fixed SAM concentration of 4 mM. Michaelis–Menten constants (Km) and catalytic constants (Kcat) were calculated using non-linear regression analysis.

### Functional validation in tea plants

For *CsNOMT* and *CsMYC2.1* overexpression, gene was constructed into the pCAMBIA302 vector and transferred into tea plants (Agrobacterium-mediated transformation). The Software for Statistical Folding of Nucleic Acids (https://sfold.wadsworth.org/cgi-bin/index.pl) was used to build AsODNs for gene silencing. Tea leaves were infiltrated with 20 μM of the sense-strand controls, CsNOMT-specific AsODNs, CsMYC2.1-specific AsODNs. One apical bud and two nearly leaves were collected from plant samples (n = 6 biological replicates) 24 hours post-treatment. Samples were utilized to assess the amount of SAK and analyze gene expression.

### Yeast one-hybrid assay

For yeast one-hybrid (Y1H) tests, *CsNOMT* promoter fragments from both cultivars (LJ43 and ZJ) were cloned into the pHIS2.1, while the coding sequence of CsMYC2.1 was ligated into pGADT7. *CsNOMT* promoter-pHIS2.1 were co-transformed into Y187 yeast cells alongside either the CsMYC2.1-AD prey construct or an empty pGADT7 control using a synthetic dropout medium lacking histidine, leucine, and tryptophan (-His/-Leu/-Trp), containing increasing concentrations of 3-amino-1,2,4-triazole (3-AT).

### Dual-luciferase assay

An effector plasmid was introduced into tobacco cells together with the reporter plasmid to express the target gene CsMYC2.1. The reporter plasmid contained the firefly luciferase gene (LUC), which was controlled by the *CsNOMT* promoter fragment from both cultivars (LJ43 and ZJ). Renilla luciferase (REN), which was constitutively produced under a constitutive promoter, served as the internal control. Luciferase activity was evaluated 48 to 72 hours after infiltration using the dual-Luciferase Reporter Assay System (Promega, USA). Each sample's firefly luciferase activity was normalized to the corresponding Renilla luciferase activity (reported as LUC/REN ratio). The transactivation potency of CsMYC2.1 on the *CsNOMT* promoter was assessed by comparing the experimental group co-expressing CsMYC2.1 to the control group, which containing just the empty vector.

### Statistical analysis

Biological were conducted in triplicate for all experiments. Results are expressed as mean values ± standard error (SE). Statistical significance was determined by Student's t-test (pairwise comparisons) or one-way ANOVA with Tukey's *post hoc* analysis (multiple comparisons), with significance thresholds set at ^**^P < 0.01 and ^*^P < 0.05. Data analyses were executed using GraphPad Prism 8.0.

## Supplementary Material

Web_Material_uhag022

## Data Availability

All the data supporting the findings of this study are available in the paper and supplementary data.
