# Supplementary figures and images for: The JA–CsMYC2.1–CsNOMT–sakuranetin module contributes to differential anthracnose resistance in Camellia sinensis
Source: Hortic Res. 2026 Jan 29;13(5):uhag022. doi: 10.1093/hr/uhag022 (PMC13143377; doi:10.1093/hr/uhag022)

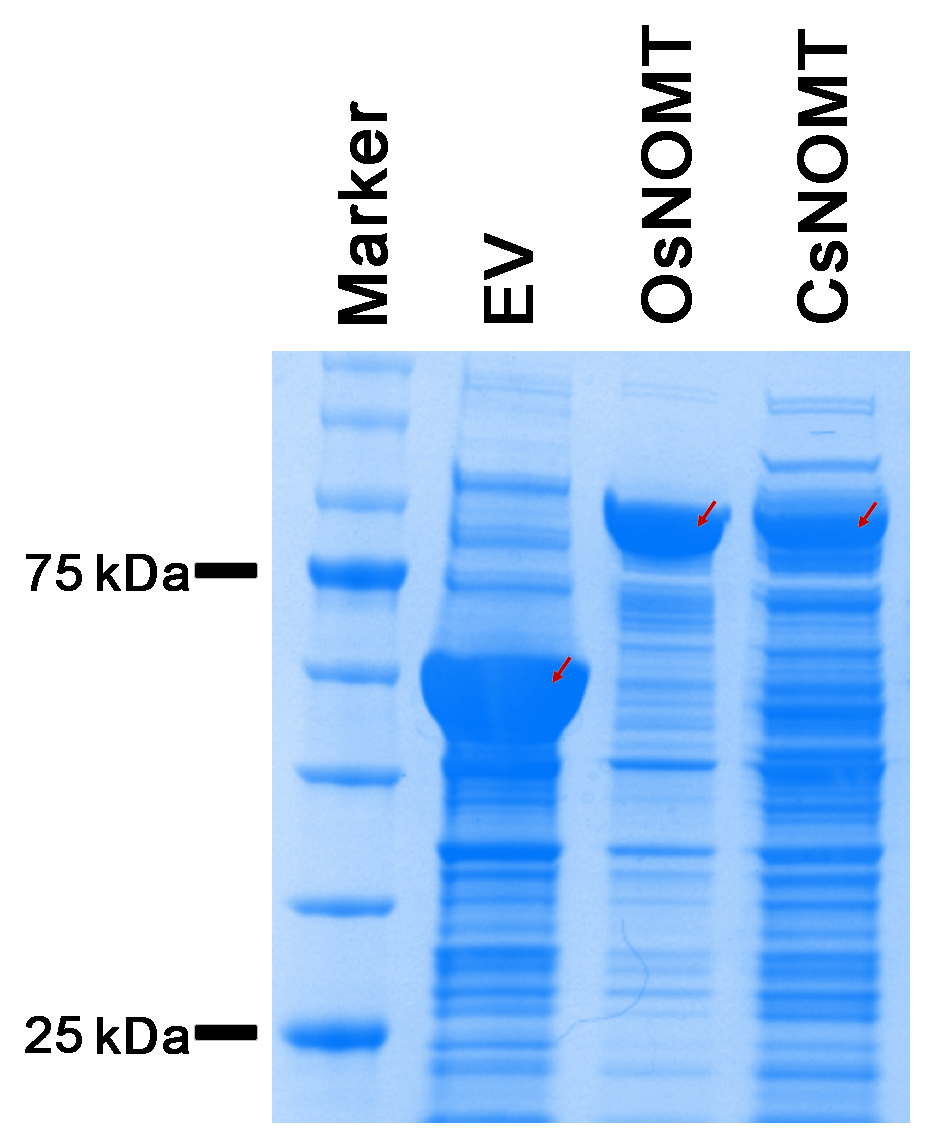

Supplement: Web_Material_uhag022 [file web_material_uhag022.zip › Fig S1.tif]

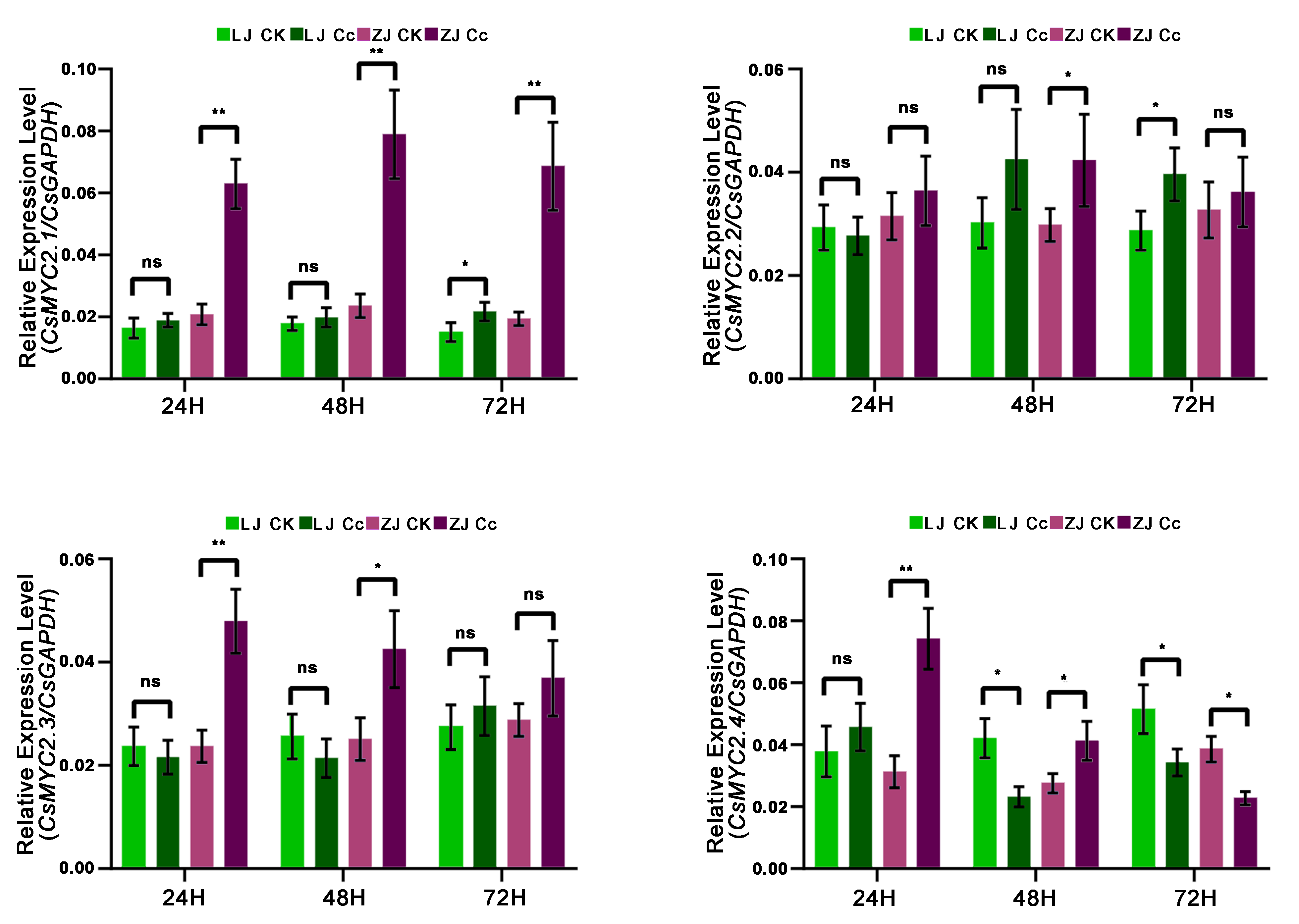

Supplement: Web_Material_uhag022 [file web_material_uhag022.zip › Fig S2.tif]

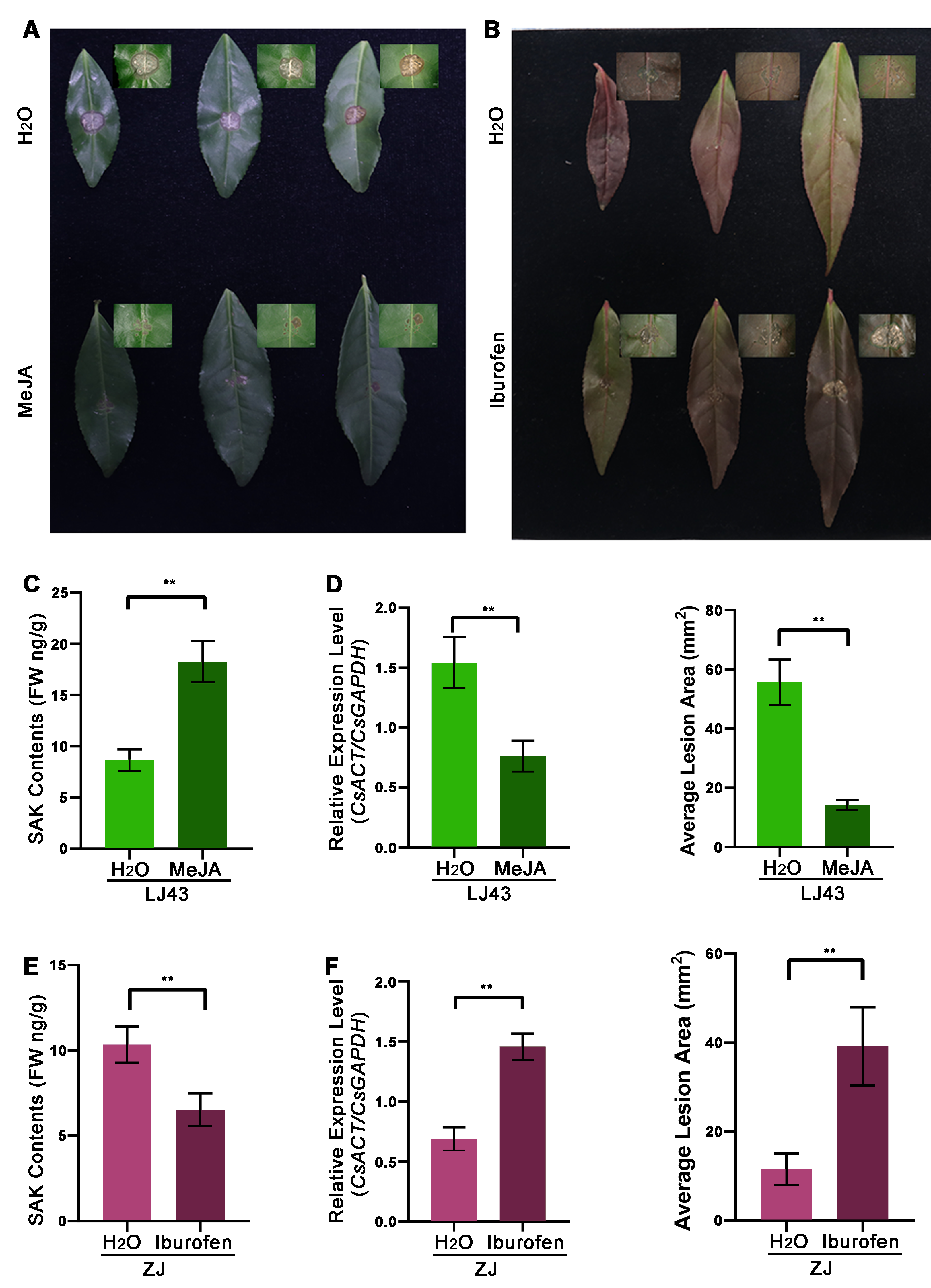

Supplement: Web_Material_uhag022 [file web_material_uhag022.zip › Fig S3.tif]

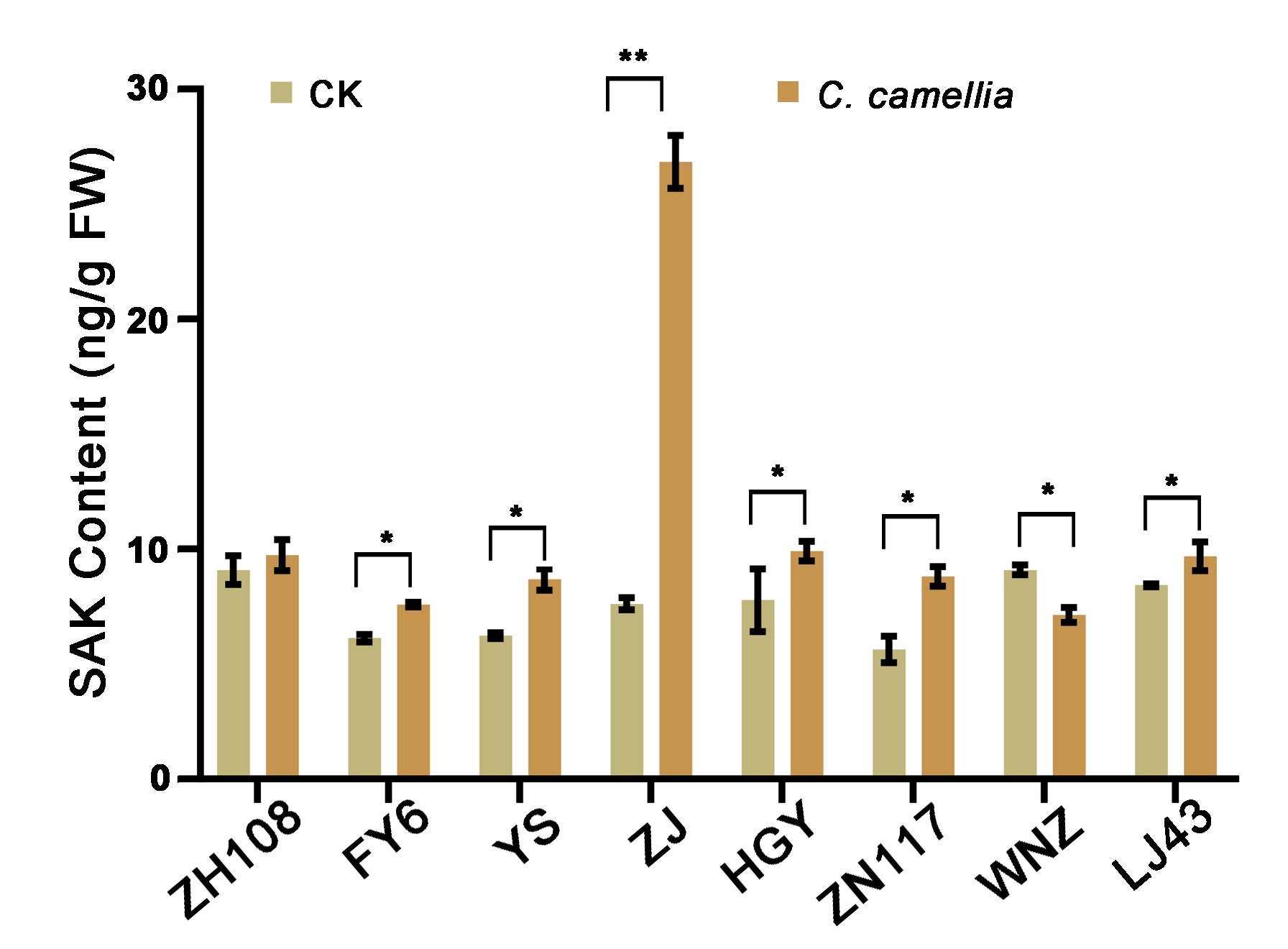

Supplement: Web_Material_uhag022 [file web_material_uhag022.zip › Fig S4.tif]

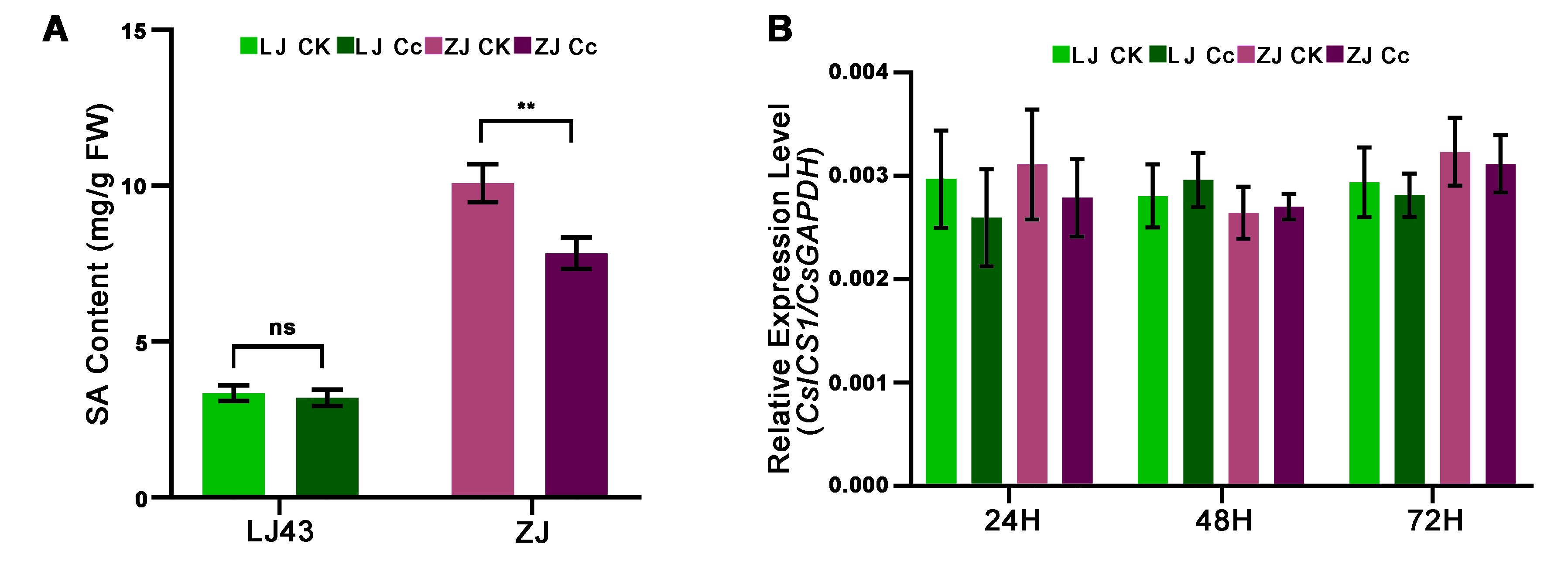

Supplement: Web_Material_uhag022 [file web_material_uhag022.zip › Fig S5.tif]
